# Supplementary material for: Ambient temperature exposure on inflammatory blood proteins—A longitudinal self-sampling survey in young Swedish adults
Source: iScience. 2025 Dec 8;29(1):114383. doi: 10.1016/j.isci.2025.114383 (PMC12800436; doi:10.1016/j.isci.2025.114383)
Supplement: Document S1. Figures S1–S9 and Tables S1–S3 and S5 [file mmc1.pdf]

iScience, Volume *29*

Supplemental information

Ambient temperature exposure on inflammatory blood proteins—A longitudinal self-sampling survey in young Swedish adults

Zhebin Yu, Sophia Björkander, Annika Bendes, Federica Nobile, Jiawei Zhang, Simon Kebede Merid, Natalia Hernandez-Pacheco, Shizhen He, Maura M. Kere, Susanna Klevebro, Petter Ljungman, Massimo Stafoggia, Tom Bellander, Göran Pershagen, Anna Bergström, Inger Kull, Anne-Sophie Merritt, Niclas Roxhed, Olena Gruzieva, Jochen M. Schwenk, and Erik Melén

Table S1 Comparison of the basic characteristics of the participants included in this study,all participants of the 24-year follow-up, and subjects that contributed to the Phase 1 of the COVID-19 follow-up.

Characteristics	Included participants (N=807)	All participants that attended the 24-year clinical examination (N=2270)	Phase 1 questionnaire (N=1664)
Sex, n (%)			
Male	291 (36.1)	1004 (44.2)	648 (39.4)
Female	516 (63.9)	1226 (55.8)	996 (60.6)
Age at 24-year follow-up, years, mean±sd	22.4 ± 0.4	22.4±0.5	22.4±0.5
Education level at 24-year , n (%)			
Elementary school/high school	474 (58.7)	1402 (61.8)	997 (60.6)
University/college	333 (41.3)	859 (37.8)	647 (39.4)
Occupation status, n (%)			
Study	454 (56.3)	1201 (52.9)	907 (55.2)
Empolyed	303 (37.5)	890 (39.2)	628 (38.2)
Other	50 (6.2)	176 (7.8)	109 (6.6)
Body mass index, kg/m2, mean±sd	22.8±3.8	23.1±3.9	23.0±3.8
Current smoking, n (%)	142 (17.6)	457 (20.1)	311 (18.9)
Asthma, n (%)	110 (13.6)	333 (14.7)	241 (14.7)
Lung function at 24-year follow-up			
FEV1, L	3.9±0.8	4.0±0.8	4.0±1.0
FVC, L	4.7±1.0	4.9±1.1	4.9±1.1

Table S2 Full list of the proteins significantly associated with temperature exposure.

Gene name	Protein Name	Function*	FDR adjusted P values
NFATC1	Nuclear factor of activated T-cells, cytoplasmic 1	Transcription factor involved in T cell activation	4.06E-29
IL18	Interleukin-18	Proinflammatory cytokine involved in innate and adaptive immunity	2.51E-27
EGF	Pro-epidermal growth factor	Involved in cell growth, proliferation, and differentiation	2.89E-20
SHMT1	Serine hydroxymethyltransferase, cytosolic	Involved in one-carbon metabolism	1.30E-18
FOXO1	Forkhead box O1	Involved in apoptosis, stress resistance, and metabolism	2.71E-13
NT5C3A	Cytosolic 5'-nucleotidase 3A	Involved in nucleotide metabolism	2.71E-13
CD40LG	CD40 ligand	Crucial for T cell-dependent immune responses	2.71E-13
C1QA	Complement C1q subcomponent subunit A	Involved in the classical complement pathway	1.45E-12
AXIN1	Axin-1	Negative regulator of the Wnt signaling pathway	5.11E-12
PREB	Prolactin regulatory element-binding protein	Regulates prolactin gene expression	1.40E-08
LTBR	Tumor necrosis factor receptor superfamily member 3	Involved in lymphoid organ development and immune responses	3.25E-08
ATP5IF1	ATPase inhibitor, mitochondrial	Regulates mitochondrial ATP synthesis	3.39E-08
EIF5A	Eukaryotic translation initiation factor 5A-1	Promotes translation elongation and termination	4.34E-08
METAP1D	Methionine aminopeptidase 1D	Involved in protein modification and processing	8.74E-08
CTSC	Dipeptidyl peptidase 1	involved in protein degradation and immune cell activation	1.83E-07
PRDX5	Peroxiredoxin-5, mitochondrial	involved in cellular antioxidant defense	3.04E-07
TNFRSF4	Tumor necrosis factor receptor superfamily member 4	involved in T cell activation and survival	6.65E-07
SPRY2	Protein sprouty homolog 2	Negatively regulates receptor tyrosine kinase signaling	7.00E-07
PSMG3	Proteasome assembly chaperone 3	Involved in proteasome assembly	7.29E-07
FXYD5	FXYD domain-containing ion transport regulator 5	Modulates Na+/K+-ATPase function	9.46E-07
FIS1	ATP synthase subunit beta	Role in ATP production within mitochondria	1.28E-06
BSG	Basigin	Involved in cell adhesion and signaling	1.40E-06
SIT1	Signaling threshold-regulating transmembrane adapter 1	T-cell receptor signaling	3.63E-06

MANF	Mesencephalic astrocyte-derived neurotrophic factor	Involved in neuroprotection and ER stress response	9.83E-06
F2R	Proteinase-activated receptor 1	Involved in platelet activation and inflammation	1.41E-05
BCR	Breakpoint cluster region protein	Involved in signal transduction and GTPase activation	4.32E-05
MAPK9	Mitogen-activated protein kinase 9	Involved in stress response and apoptosis	8.07E-05
TGFB1	Transforming growth factor beta-1 proprotein	Involved in cell growth, differentiation, and immune regulation	9.08E-05
GOPC	Golgi-associated PDZ and coiled-coil motif-containing protein	Involved in intracellular protein trafficking	0.000585764
ANGPTL2	Angiopoietin-related protein 2	Involved in angiogenesis and inflammation	0.000740986
MATN2	Matrilin-2	Involved in extracellular matrix assembly	0.000740986
SPINT2	serine peptidase inhibitor, Kunitz type 2	Encodes a protease inhibitor protein that acts as a tumor suppressor and regulates epithelial functions.	0.000766367
TPT1	Translationally-controlled tumor protein	Involved in calcium binding and microtubule stabilization	0.001383389
BTN3A2	Butyrophilin subfamily 3 member A2	Involved in T cell activation	0.001701785
HPCAL1	Hippocalcin-like protein 1	Primarily in calcium signaling within neurons, impacting processes related to vision and neuronal communication	0.002257921
LAMP3	Lysosome-associated membrane glycoprotein 3	Involved in dendritic cell function and antigen presentation	0.002703022
CHRDL1	Chordin-like protein 1	Involved in bone morphogenetic protein signaling regulation	0.005040984
TGFA	Protransforming growth factor alpha	Involved in cell proliferation and differentiation	0.005761049
AGRP	Agouti-related protein	Involved in appetite regulation	0.005885621
LSP1	Lymphocyte-specific protein 1	Involved in neutrophil transendothelial migration	0.006679847
MEPE	Matrix extracellular phosphoglycoprotein	Involved in bone mineralization	0.008031176
ADGRE2	Adhesion G protein-coupled receptor E2	Involved in immune cell adhesion and activation	0.008031176
TNFRSF13B	Tumor necrosis factor receptor superfamily member 13B	Involved in B cell activation and survival	0.010228565
SKAP2	Src kinase-associated phosphoprotein 2	Plays a vital role as an adaptor protein in immune signaling, particularly in the regulation of integrin-mediated functions in neutrophils and macrophages	0.010228565
IKBKG	NF-kappa-B essential modulator	Essential for activating NF-kappa B	0.01154277

LAP3	Cytosol aminopeptidase	Involved in protein degradation and processing	0.011668569
IL32	Interleukin-32	Proinflammatory cytokine involved in innate and adaptive immune responses	0.017452406
NBN	Nibrin	Crucial for DNA double-strand break repair and maintenance of chromosome integrity	0.019944241
TRIM21	tripartite motif-containing protein 21	Role in linking the adaptive immune response (through antibodies) with innate immune mechanisms, primarily through its functions as an intracellular antibody receptor and E3 ubiquitin ligase	0.022355331
SIGLEC1	Sialoadhesin	Involved in cell-cell interactions and immune response	0.026606549
CD40	Tumor necrosis factor receptor superfamily member 5	Pivotal receptor in the immune system, facilitating critical interactions between T cells and B cells, promoting antibody production, and regulating immune responses.	0.026606549
BANK1	B-cell scaffold protein with ankyrin repeats	Involved in B cell receptor signaling	0.028943906
PIK3AP1	Phosphoinositide 3-kinase adapter protein 1	Plays a crucial role in promoting the activation of phosphoinositide 3-kinase (PI3K) signaling	0.029586984
MPIG6B	Megakaryocyte And Platelet Inhibitory Receptor G6b	Involved in the immune response, particularly in the regulation of T cell activation and function.	0.029586984
SCGB1A1	Secretoglobin Family 1A Member 1	Anti-inflammatory proteins that highly expressed in airway and lung	0.336257385
DAG1	Dystroglycan 1	Essential for the structural integrity of muscle cells and other tissues. It serves as a receptor for extracellular matrix components and is involved in cell adhesion, signaling, and the maintenance of muscle fiber integrity.	0.040481317
CXCL1	Chemokine (C-X-C motif) ligand 1	Acts by binding to its receptor CXCR2, promoting chemotaxis and activation of neutrophils, thereby contributing to the body's defense mechanisms against pathogens.	0.046383676
CSF1	Macrophage colony-stimulating factor 1	Involved in macrophage differentiation and function	0.049179281

* Function according to The Human Protein Atlas (https://www.proteinatlas.org/).

**: P values derived from the likelihood test comparing the model with and without temperature exposure

Table S3 Associations between inflammation-related proteins linked to temperature and clinical phenotypes in the BAMSE study and UKB-PPP.

Protein name	BAMSE			UKB-PPP			
	Clinical phenotype	Association	P-value	Clinical phenotype	ICD 10	Association	P-value
DNER	FEV1/FVC	Beta=0.008	0.003	Pneumonia organism unspecified	J18	OR=0.88	1.52E-10
				Unspecified acute lower respiratory infection	J22	OR=0.92	2.20E-08
				Other chronic obstructive pulmonary disease	J44	OR=0.87	1.40E-08
TGFB1	FEV1	Beta= -0.039	0.026	Other chronic obstructive pulmonary disease	J44	OR=1.24	4.58E-13
				Pneumonia organism unspecified	J18	OR=1.16	1.20E-18
				Respiratory failure	J96	OR=1.27	1.63E-08
TNFRSF13B	FEV1/FVC	Beta=-0.007	0.031	Pneumonia organism unspecified	J18	OR=1.19	2.51E-17
				Unspecified acute lower_respiratory_infection	J22	OR=1.10	4.88E-11
				Emphysema	J43	OR=1.31	8.27E-08
				Other chronic obstructive_pulmonary_disease	J44	OR=1.23	9.67E-18
				Pulmonary edema	J81	OR=1.81	3.91E-08
				Respiratory failure not_elsewhere_classified	J96	OR=1.32	1.18E-11
TNFRSF4	FEV1/FVC	Beta=-0.006	0.024	Pneumonia organism unspecified	J18	OR=1.31	9.93E-39
				Unspecified acute lower_respiratory_infection	J22	OR=1.12	1.81E-14
				Emphysema	J43	OR=1.38	1.19E-10
				Other chronic obstructive_pulmonary_disease	J44	OR=1.34	1.37E-33
				Bronchiectasis	J47	OR=1.34	1.20E-10
				Pleural effusion not_elsewhere_classified	J90	OR=1.24	7.49E-13
				Respiratory failure not_elsewhere_classified	J96	OR=1.44	6.86E-19
SCGB1A1	FEV1/FVC	Beta=0.07	0.018	Unspecified acute ower_respiratory_infection	J22	OR=0.91	1.12E-10
				Emphysema	J43	OR=0.57	1.10E-30
				Other chronic bstructive_pulmonary_disease	J44	OR=0.58	7.55E-111
				Asthma	J45	OR=0.82	8.99E-42
SCGB1A1	SBP	-1.69	0.001	SBP		Beta=-0.07	3.31E-30
SCGB1A1	DBP	-0.94	0.030	DBP		Beta=-0.06	1.16E-39
BANK1	DBP	Beta=0.76	0.048	DBP	NA	Beta=0.05	3.34E-24

NFATC1	DBP	Beta=0.877	0.018	DBP	NA	Beta=0.047	1.79E-21
TNFRSF4	DBP	Beta=-0.95	0.007	DBP	NA	Beta=-0.028	1.15E-08
C1QA	HbA1c	Beta=-0.26	0.018	HbA1c	NA	Beta=-0.025	1.70E-07
MEPE	HbA1c	Beta=-0.25	0.039	HbA1c	NA	Beta=-0.395	1.19E-16
SCGB1A1	HbA1c	Beta=-0.295	0.026	HbA1c	NA	Beta=-0.05	4.44E-20

FEV1: forced expiratory volume in 1 second, FVC: forced vital capacity, SBP: systolic blood pressure, DBP: diastolic blood pressure, HbA1c: hemoglobin A1c. UKB-PPP: UK biobank Pharma Proteomic Project

Table S5 Importance of the final features in the lightGBM model

Feature (protein name)	Gain	Cover	Frequency
RABGAP1L	0.237094	0.049838	0.02998544
PLA2G4A	0.039927	0.035427	0.03187773
CD22	0.038021	0.040355	0.02809316
EIF4G1	0.036996	0.015538	0.01717613
TIMP3	0.036085	0.018457	0.01935953
PIK3AP1	0.03564	0.014893	0.01615721
MAP2K6	0.030943	0.03264	0.03042213
IRAK4	0.029144	0.02109	0.02634643
GLOD4	0.023088	0.035979	0.0294032
NFATC3	0.02274	0.01893	0.02139738
SPON1	0.021886	0.018455	0.02212518
PRKAB1	0.021572	0.021999	0.02561863
DBNL	0.020131	0.014108	0.01804949
TBC1D5	0.019902	0.019294	0.02299854
NUB1	0.019716	0.034147	0.02576419
TLR3	0.019003	0.017753	0.02212518
TRAF2	0.017006	0.016523	0.02445415
ANGPTL2	0.016313	0.014467	0.0202329
PLXNA4	0.015862	0.024991	0.02139738
JUN	0.014733	0.019044	0.02183406
DAPP1	0.014687	0.035403	0.02518195
CKMT1A_CKMT1B	0.013523	0.026043	0.02212518
NT5C3A	0.012841	0.027118	0.02066958
NUDC	0.012175	0.0177	0.01834061
CTRC	0.01199	0.030786	0.02343523
ARHGEF12	0.011988	0.021804	0.02212518
PPP1R9B	0.011975	0.019099	0.01877729
BSG	0.011959	0.025029	0.02037846
DAG1	0.01182	0.016404	0.01979622
PSMG3	0.011653	0.021566	0.02256186
CEP164	0.011496	0.014757	0.01790393
BCR	0.011482	0.017507	0.01746725
GAL	0.011419	0.021775	0.01819505
PNPT1	0.010517	0.012895	0.01965066
EIF5A	0.010093	0.012967	0.01848617
ACTN4	0.0094	0.012644	0.01717613
CXCL17	0.009242	0.014748	0.01804949
CLSTN2	0.009054	0.011563	0.01455604
CCL22	0.009048	0.017643	0.01499272
BTN2A1	0.008831	0.023911	0.01906841
SELPLG	0.008623	0.017313	0.01906841
MYO9B	0.008617	0.019057	0.01979622
IL1RN	0.008555	0.016096	0.0224163
CD40LG	0.008471	0.010732	0.01615721
TPSAB1	0.008373	0.023949	0.02212518

TNFRSF11A	0.008308	0.017281	0.01892285
IL4R	0.008058	0.010282	0.01775837

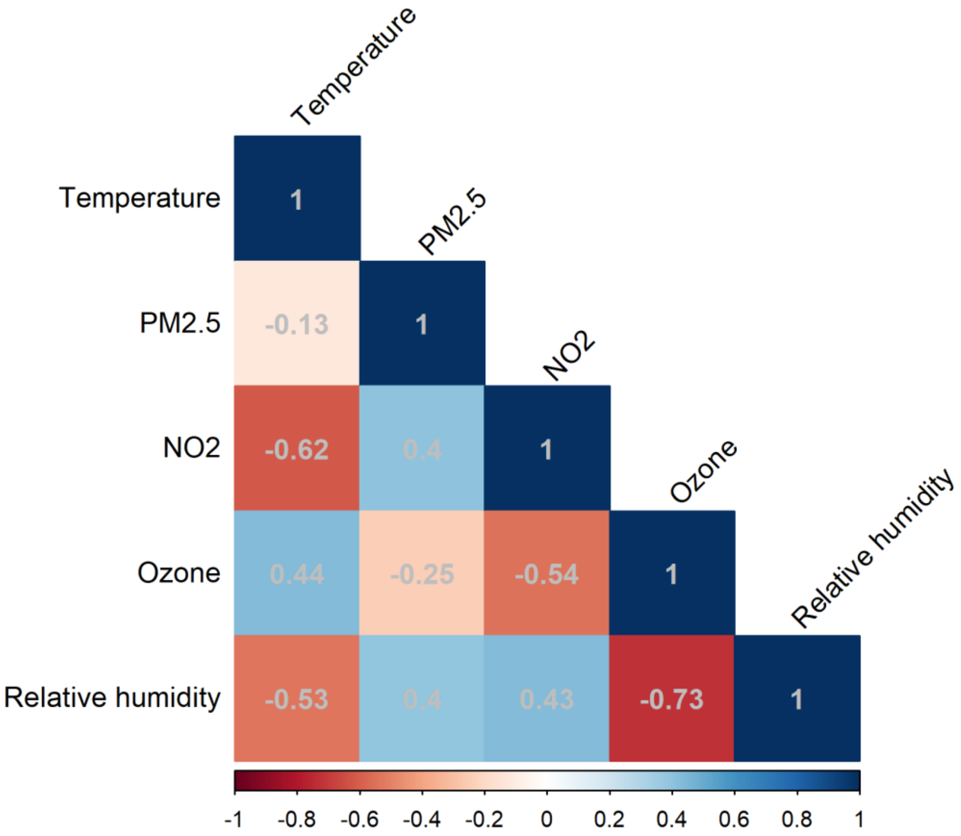

Figure S1. Spearman correlations between temperature, air pollution levels, and relative humidity. PM2.5, particulate matter with diameter ≤2.5 µm; NO2, nitrogen dioxide. The correlations were calculated on the exposure of each individual.

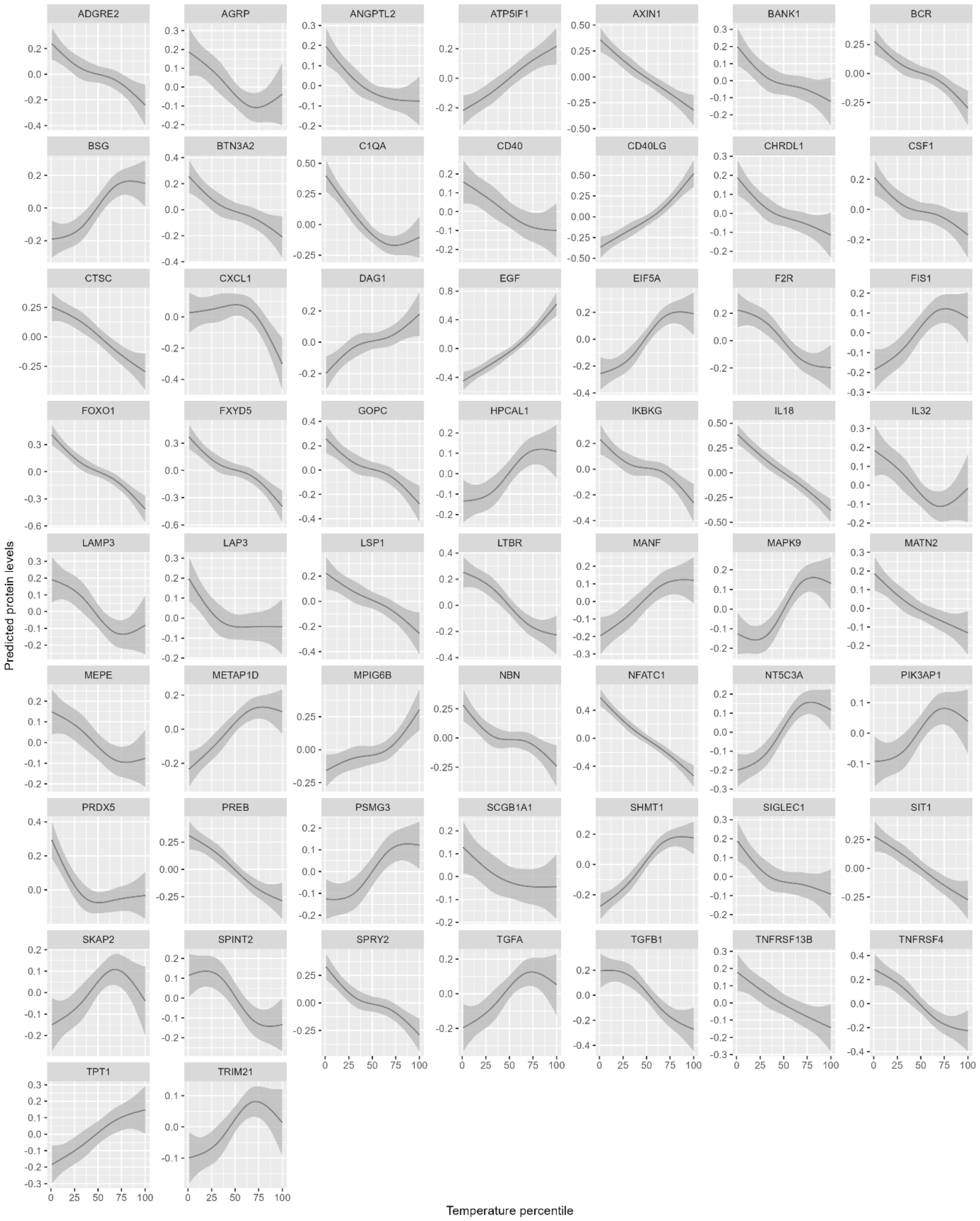

Figure S2. Exposure-response curves for proteins significantly associated with temperature. The regression model was adjusted for age, sex, long-time trend (nature spline term with six degrees of freedom), phase, day of the week, season (warm: April to September; cold: October to March), history of SARS-CoV-2 infection, and vaccination at the time of biosampling. Natural splines with four degrees of freedom were applied to explore the potential non-linear relationships.

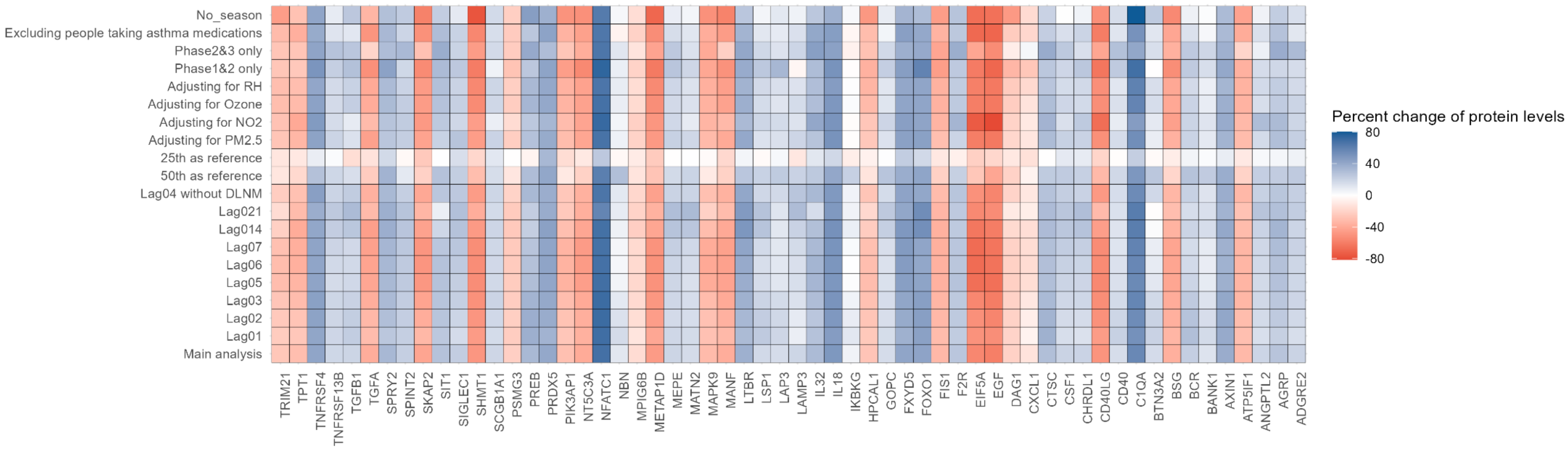

Figure S3 Sensitivity analysis on the association between extreme cold and inflammation-related proteins. All the results were adjusted for age, sex, long time trend (nature spline term with six degrees of freedom), phases, day of the week, season (warm: April to September; cold: October to March), history of SARS-CoV-2 infection and vaccination at the time of biosampling. Estimates presented in table format were provided in Table S4.

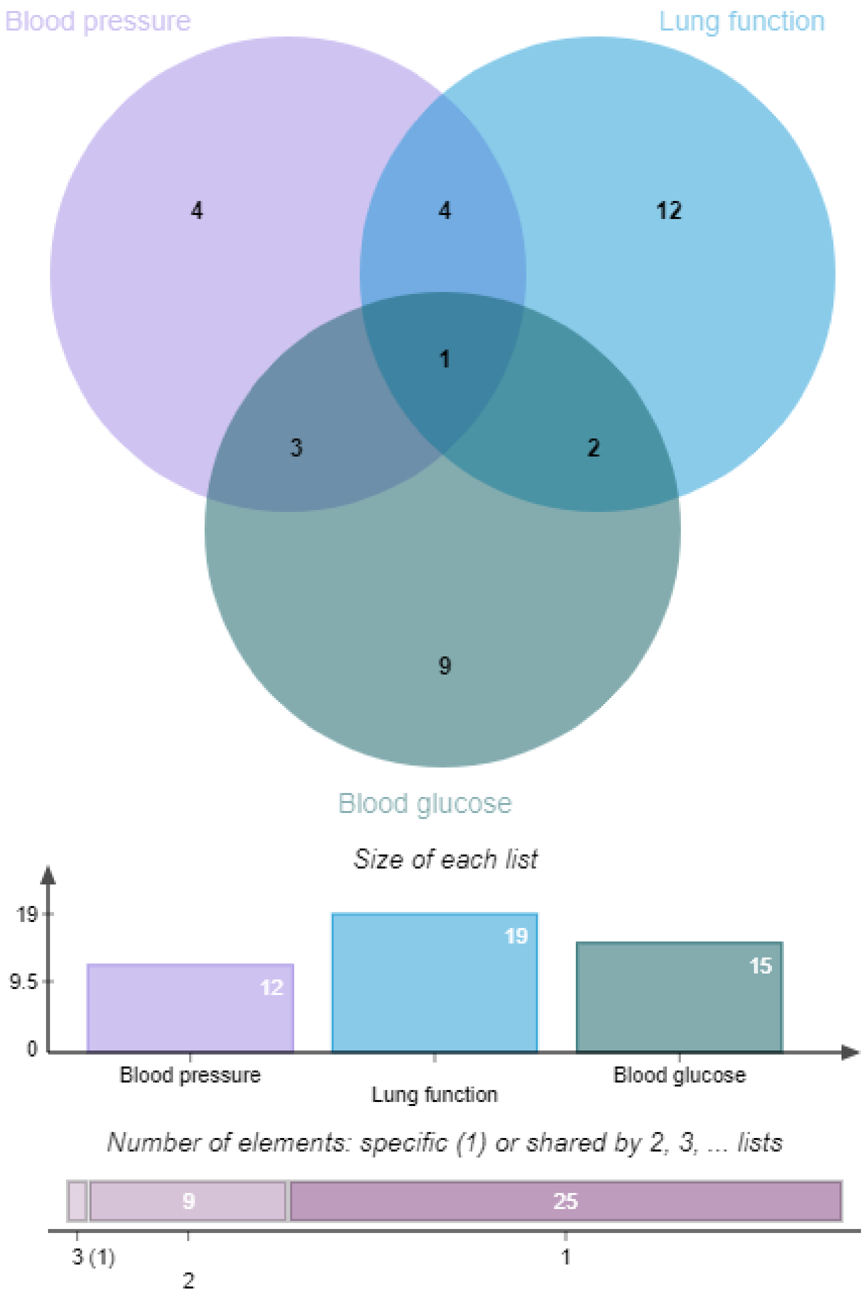

Figure S4. Overlapping inflammation-related proteins associated with lung function, blood pressure, and HbA1c. Only the proteins significantly associated with temperature exposure are shown.

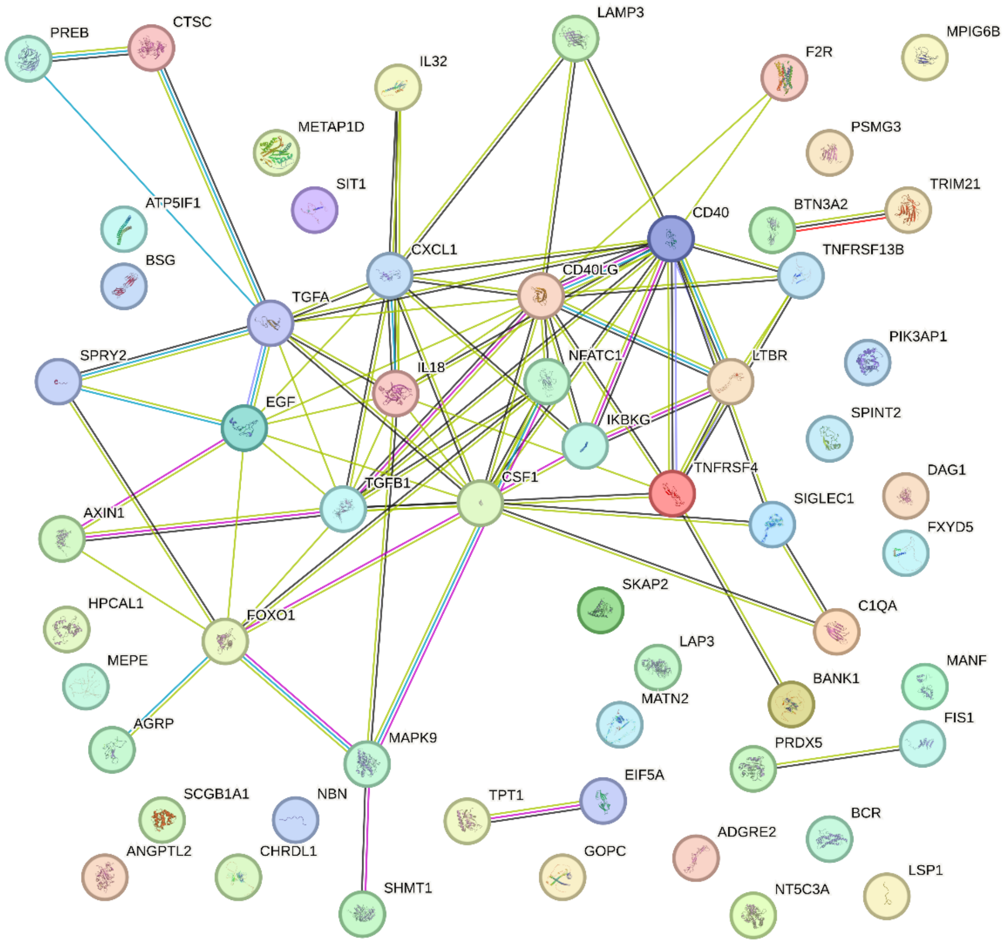

Figure S5 Results of the protein-protein interaction network analysis on all proteins associated with temperature

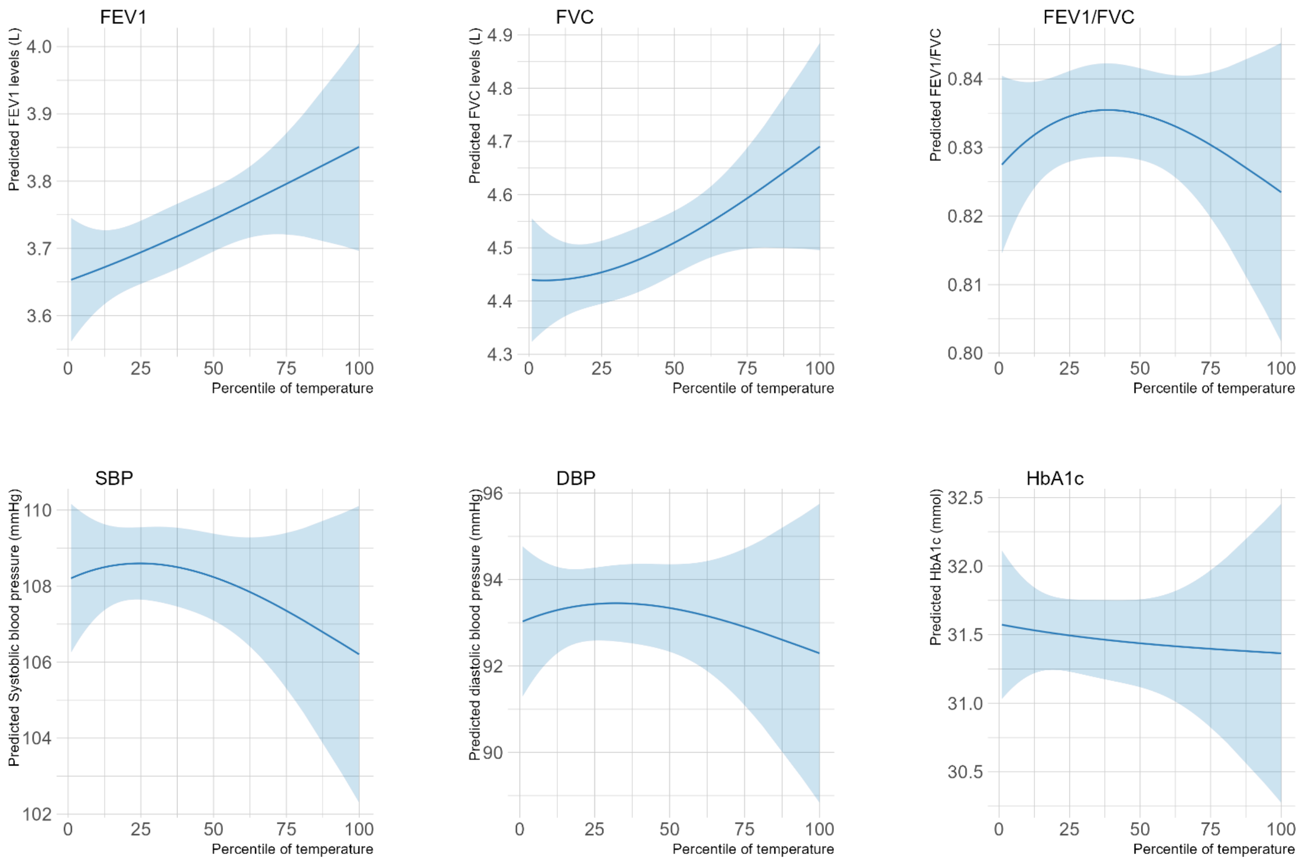

Figure S6. Cross-sectional association results between short-term exposure to temperature (lag0-4) and lung function, blood pressure, and HbA1c. Nature splines with two degrees of freedom were applied to explore the potential non-linear relationship. Results were adjusted for age, sex, height (for lung function), or body mass index (for blood pressure and HbA1c). FEV1, forced expiratory volume in 1 second; FVC, forced vital capacity; SBP, systolic blood pressure; DBP, diastolic blood pressure.

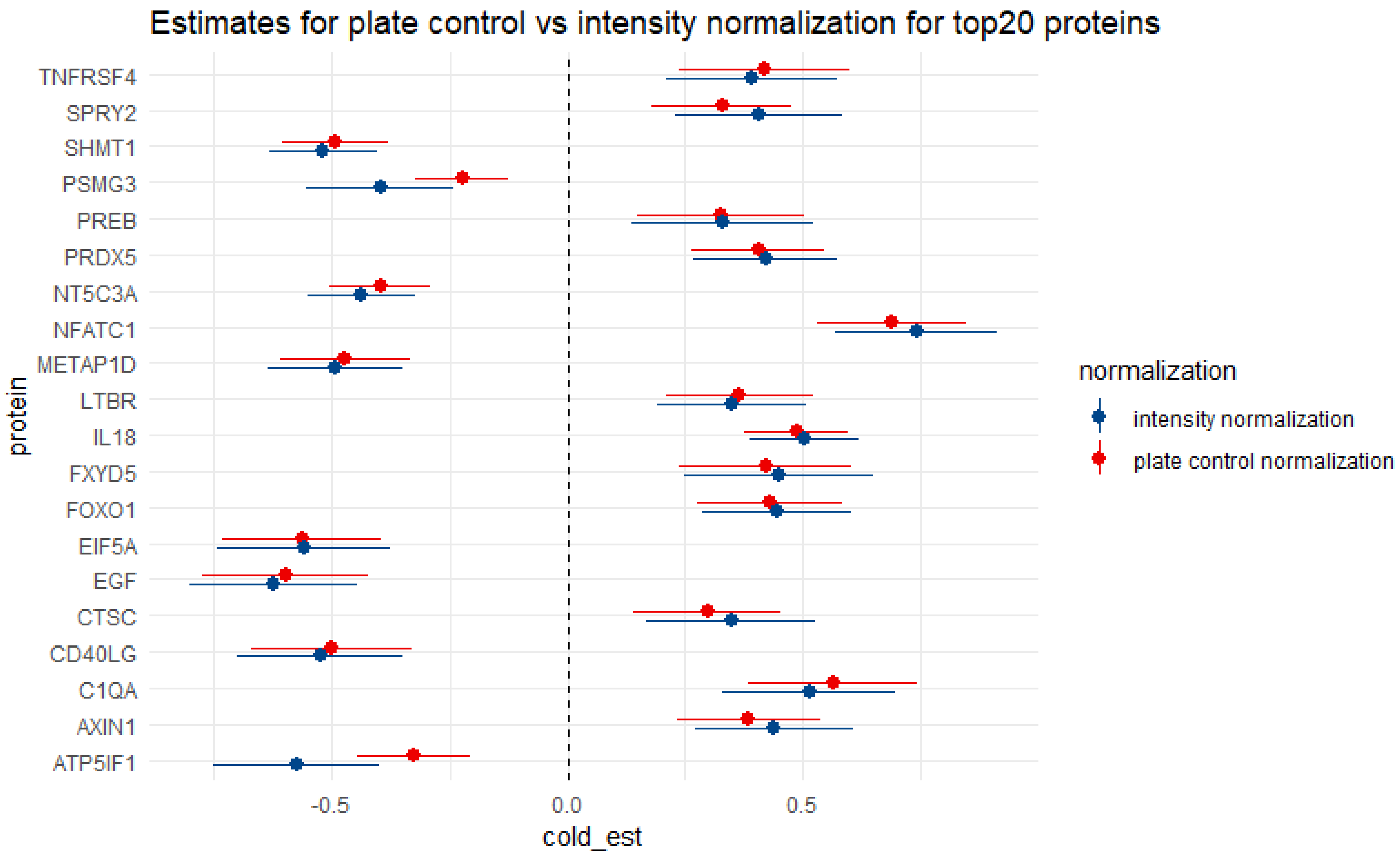

Figure S7 Comparison of association estimates of extreme cold exposure with top20 inflammation-related proteins using the plate control normalization and intensity normalization method. All the results were adjusted for age, sex, long time trend (nature spline term with six degrees of freedom), phases, day of the week, season (warm: April to September; cold: October to March), history of SARS-CoV-2 infection and vaccination at the time of biosampling

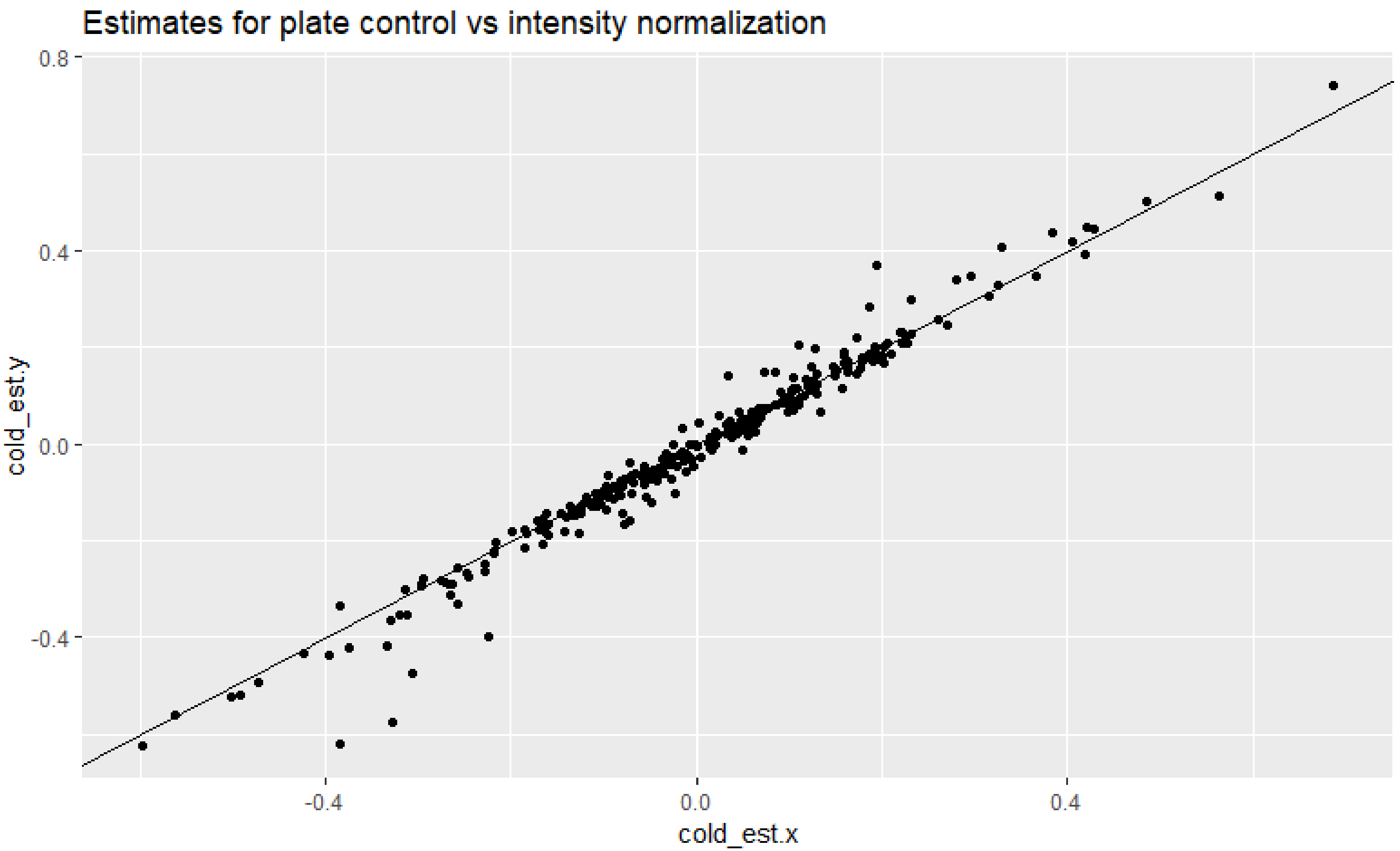

Figure S8 Comparison of association estimates of extreme cold exposure with all the inflammation-related proteins using the plate control normalization and intensity normalization method. All the results were adjusted for age, sex, long time trend (nature spline term with six degrees of freedom), phase of follow-up, day of the week, season (warm: April to September; cold: October to March), history of SARS-CoV-2 infection and vaccination at the time of follow-ups

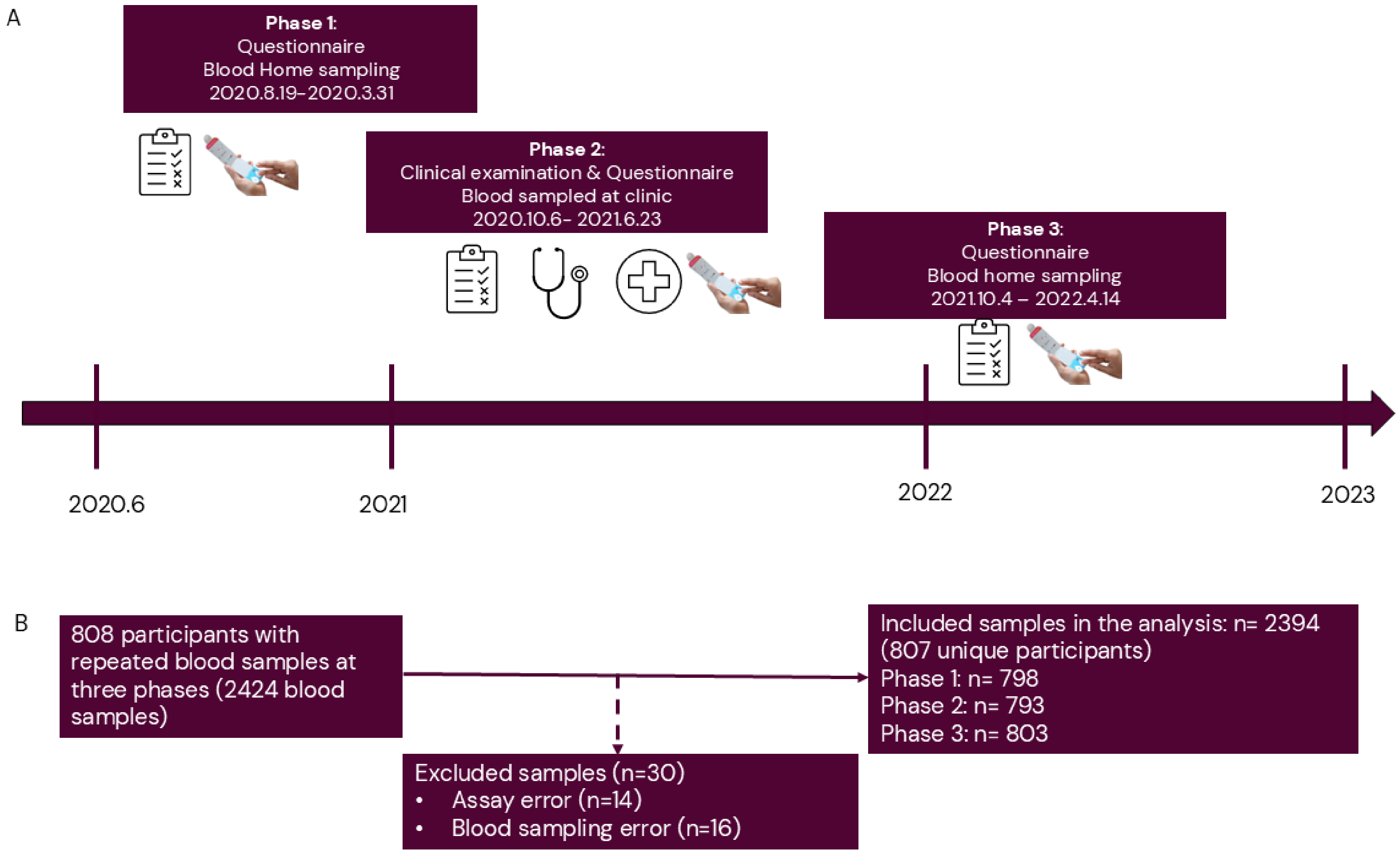

Figure S9. (A) Timeline of the COVID-19 follow-ups of the BAMSE study (B) Flow chart of the included blood samples.
